# Climate Change Adaptation in Winemaking: Combined Use of Non-*Saccharomyces* Yeasts to Improve the Quality of Pedro Ximénez Wines

**DOI:** 10.3390/microorganisms13081908

**Published:** 2025-08-15

**Authors:** Fernando Sánchez-Suárez, Rafael Martínez-García, Rafael A. Peinado

**Affiliations:** Agricultural Chemistry, Soil Science and Microbiology Department, University of Córdoba, Campus of Rabanales, N-IV Road, Km 396, 14071 Córdoba, Spain; g62sasuf@uco.es (F.S.-S.); q72magar@uco.es (R.M.-G.)

**Keywords:** *L. thermotolerans*, *M. pulcherrima*, Pedro Ximénez, climate change, acidity, lactic acid, volatile compounds

## Abstract

This study evaluates the impact of two non-*Saccharomyces* yeasts, *Lachancea thermotolerans* and *Metschnikowia pulcherrima*, on the oenological and sensory characteristics of Pedro Ximénez (PX) white wines produced in warm regions of southern Spain. PX wines are particularly affected by climate change, often exhibiting low acidity and limited aromatic complexity. Fermentations were performed using pure and sequential cultures of these yeasts and compared to a control inoculated with *Saccharomyces cerevisiae*. Wines fermented with *L. thermotolerans* showed increased titratable acidity (up to 6.83 g/L), reduced pH (down to 3.02), and higher lactic acid concentrations, contributing to improved freshness and microbial stability. The use of *M. pulcherrima* led to a significant rise in ester production, enhancing fruity and floral aromatic notes. Sequential fermentation using both yeasts produced wines with the highest overall aromatic complexity and superior performance in sensory evaluations. These findings support the use of *L. thermotolerans* and *M. pulcherrima* as a promising biotechnological strategy to improve white wine quality under climate change conditions.

## 1. Introduction

The wine industry is currently facing significant challenges due to the accelerating effects of climate change [1]. This is particularly evident in warm regions such as the Montilla-Moriles area of Andalusia in southern Spain, where the main grape variety is Pedro Ximénez (PX).

Although PX is a grape variety adapted to dry climates, negative consequences of climate change are expected in the medium term, which will affect both production and wine quality. Rising global temperatures and increased exposure to sunlight mean that grapes reach maturity earlier because they accumulate sugars faster than acids are degraded. This reduces total acidity and malic acid content [1,2]. However, one of the most significant effects of global warming on grapevine physiology is the separation of sugar and phenolic or aromatic maturity [3]. Consequently, high berry temperatures hinder the production of certain terpenoids and norisoprenoids, while accelerating the breakdown of anthocyanins and volatile precursors [4,5].

These changes result in wines with a higher alcohol content, as well as lower freshness, aromatic complexity, and ageing potential. Technological corrections are required to preserve both typicity and consumer preferences. However, some traditional winemaking solutions, such as acidification (e.g., adding tartaric acid) and reducing alcohol through physical methods (e.g., spinning cone or reverse osmosis), are still used. The use of selected *Saccharomyces cerevisiae* strains is also limited in terms of cost, sensory impact, and consumer perception [6].

Recent trends in winemaking have focused on utilising a combination of *Saccharomyces* and non-*Saccharomyces* yeasts. This approach is considered a promising, natural, and sustainable way of reducing the negative impact of climate change on wine quality [6,7,8,9,10]. Notably, *Lachancea thermotolerans* produces substantial amounts of lactic acid. Depending on the strain and must composition, this yeast has been observed to reduce final pH values by up to 0.4 units and increase titratable acidity by 1.5–3 g/L [6,9,11]. These changes have the potential to enhance the wine’s perceived freshness and microbial stability without compromising its aromatic purity [9,12,13].

Over the past decade, several studies have examined the impact of *L. thermotolerans* and other non-*Saccharomyces* yeast strains, such as *Metschnikowia pulcherrima*, *Torulaspora delbrueckii*, and *Hanseniaspora vineae*, on wine quality [8,9,14,15]. Most studies have demonstrated that using these yeast species has a positive effect on the mouthfeel and sensory characteristics of wine. This is not only due to changes in acidity, but also to reductions in ethanol content and improvements in colour and glutathione production [9,16]. It is also well known that these yeasts exhibit enzymatic activities (e.g., β-glucosidase and esterase) that favour the liberation of glycosidically bound aroma precursors [9,17,18].

In this context, several research groups have examined the use of *L. thermotolerans* alongside other non-*Saccharomyces* species to produce red and white wines. These studies have reported improvements in most cases [6,9,17,18,19,20,21,22,23,24]. However, existing research focuses on aromatic grape varieties, with studies of neutral aromatic varieties mainly addressing the most widely cultivated varieties in the country [8,11,14,15,25]. There is limited research focusing on local varieties with neutral aromatic characteristics whose quality and production are compromised by cultivation in warm semi-desert areas [7,16,26].

Only two studies focusing on improving the aromatic profile and sensory quality of PX wine using *L. thermotolerans* and other non-*Saccharomyces* species have been published. Firstly, some authors have investigated the use of two indigenous yeasts, *L. thermotolerans* (CLI-P218) and *S. cerevisiae* (CLI-219), in co- and pure cultures to ferment sun-dried PX musts [26]. Secondly, other authors used commercial non-*Saccharomyces* yeasts. This study uses *L. thermotolerans* and *M. pulcherrima* (known as Laktia™ and Primaflora^®^ VB, respectively), as well as a *S. cerevisiae* yeast strain that overproduces glutathione (Glutaferm One^®^), in pure culture on unsterilised must, to produce young PX wines [16]. In both cases, the results are promising in terms of improving the sensory quality of wines made from this grape variety. However, to the authors’ knowledge, no research has been conducted on using commercial non-*Saccharomyces* yeast strains in sequential culture with *S. cerevisiae* to produce young PX wines.

This study evaluates the effect of a commercial *L. thermotolerans* yeast on the quality of young PX wines produced on a pilot scale. To do this, fermentations using the yeast in monoculture were compared with fermentations carried out sequentially with *M. pulcherrima* and/or *S. cerevisiae*. This study evaluates its impact on physicochemical parameters, the aromatic profile, and sensory perception.

## 2. Materials and Methods

### 2.1. Fermentation Conditions

For this study, must from the Pedro Ximénez grape variety was sourced from the Alvear, S.A. winery in Montilla, Córdoba, Spain. After harvesting, the grapes were destemmed and crushed under standard winery conditions. The resulting must had a reducing sugar content of 230 g/L, a pH of 3.3, and a titratable acidity of 4.98 g/L (expressed as tartaric acid).

**Figure 1:**
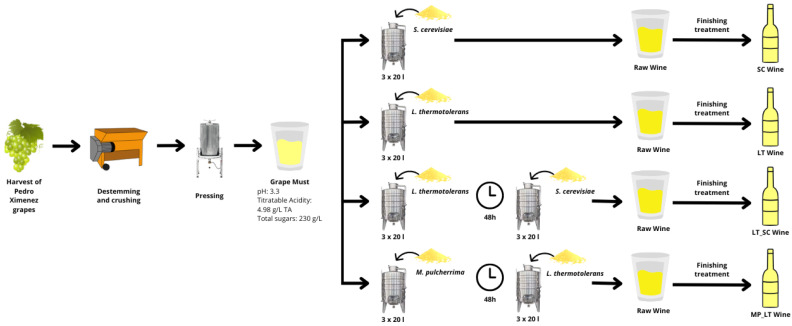
Workflow of material and methods/experimental design. TA: Tartaric acid.

Fermentations were carried out in triplicate at 17 ± 1 °C in 20-litre stainless steel tanks (Figure 1). Three commercial yeast strains were used: *L. thermotolerans* (Level 2 Laktia^®^), *M. pulcherrima* (Level 2 Flavia^®^), and *S. cerevisiae* (Opale^®^). These were supplied by Lallemand Inc. (151 Skyway Ave, Toronto, Ontario, Canada). The inoculation protocols were as follows: (1) Pure culture of *S. cerevisiae* (control); (2) Pure culture of *L. thermotolerans*; (3) *L. thermotolerans* followed by *S. cerevisiae* after 48 h after the initial inoculation; and (4) *M. pulcherrima* followed by *L. thermotolerans* after 48 h after the initial inoculation. The order of inoculations depends on the fermentation capacity of the yeasts. *L. thermotolerans* has a higher capacity than *M. pulcherrima* at 10% and 4% *v/v*, respectively, so *M. pulcherrima* was inoculated first.

In all cases, yeast was added at a dose of 25 g/hL. Fermentation progress was monitored daily by measuring density until it dropped below 995 g/L. Post-fermentation, wines were clarified with bentonite (BENGEL^®^, Agrovin S.A., Alcázar de San Juan-Ciudad Real, Spain) and gelatine (PROVEGET 100^®^, Agrovin S.A.), at doses of 25 g/hL and 15 g/hL, respectively, then cold stabilised at 3 °C.

### 2.2. Oenological Parameters

The general parameters (pH, titratable acidity, volatile acidity, ethanol content, and colour index) were analysed using official methods [27]. The concentrations of malic and lactic acids were measured by reflectometry using the Reflectoquant™ system (Merck^®^, Darmstadt, Germany).

### 2.3. Analysis of Volatile Compounds

The volatile compounds present in the must and wine were classified into two groups according to their concentration: ‘major’ volatile compounds (≥10 mg/L) and ‘minor’ volatile compounds (<10 mg/L). Three biological replicates were carried out for the analysis.

#### 2.3.1. Major Volatile Compounds and Polyols

The quantification of these compounds, including polyols, was performed using an HP 6890 Series II gas chromatograph (Agilent Technologies, Palo Alto, CA, USA), which was equipped with a CP-WAX 57 CB capillary column (50 m long with an internal diameter of 0.25 mm and a film thickness of 0.4 µm) and a flame ionisation detector (FID). This was performed in accordance with the protocol outlined by Peinado et al. [28].

For the analysis, a 10 mL wine sample was prepared with 1 mL of 4-methyl-2-pentanol (1024 mg/L) as an internal standard. An amount of 0.5 µL of this solution was then injected. Prior to injection, tartaric acid was removed by precipitation using 0.2 g of calcium carbonate, followed by centrifugation at 300 g.

The chromatographic conditions were as follows: 30:1 split ratio; FID detector; and a temperature programme starting at 50 °C for 15 min, then increasing by 4 °C/min to 190 °C (temperature maintained for 35 min). The injector temperature was 270 °C and the detector temperature was 300 °C. Helium was used as the carrier gas with an initial flow rate of 0.7 mL/min for 16 min, progressively increasing to 1.1 mL/min and maintained for 52 min. Identification and quantification were performed by injecting standards under identical conditions to those used for the samples. Table S1 contains additional information on the linear retention index (LRI) used.

#### 2.3.2. Minor Volatile Compounds

These compounds were analysed in two stages, following the method described by López de Lerma et al. [29].

In the first stage, an extraction technique involving coated stir bars (Twistter; 0.5 mm thick and 10 mm long; Gerstel GmbH, Mülheim an der Ruhr, Germany) was used. These were placed in vials containing 10 mL of diluted sample (1:10) and 0.1 mL of ethyl nonanoate (0.4464 mg/L) as the internal standard. The stir bars were shaken at 1500 rpm for 100 min, then removed and transferred to desorption tubes for further analysis.

In the second stage, the volatile compounds were analysed using a Gerstel TDS 2 thermal desorption system coupled and Agilent GC 7890A-MSD 5975 (Santa Clara, CA, USA). The stir bars were heated to 280 °C to release the volatile compounds into a refrigerated CIS 4 PTV injection system, which was programmed to 25 °C and contained an adsorption tube with Tenax. The CIS was then heated to transfer the compounds to the GC-MS, which used an Agilent 19091S capillary column (30 m × 0.25 mm internal diameter and 0.25 µm film thickness). The mass detector operated in scanning mode at 1850 V, covering a mass range of 39–300 amu.

Identification was performed by comparing the retention times of the compounds with those of analytical standards and with the Wiley spectral library. Quantification was performed using calibration curves. Further details on LRI values can be found in Table S1.

#### 2.3.3. Calculation of Aromatic Series

The Odour Activity Values (OAVs) of the volatile compounds were calculated by dividing their concentrations by their respective olfactory perception thresholds.

Aromatic series group volatile compounds with similar olfactory descriptors. The total value of each series is obtained by summing the OAVs of its compounds. Nine aromatic series were identified in total: chemical, green, citrus, creamy, floral, fruity, green fruit, honey, and waxy. Note that a volatile compound may belong to more than one series depending on its specific sensory characteristics (see Table S2).

### 2.4. Organoleptic Characterisation

A group of eight experienced judges (three men and five women) from the Department of Agricultural Chemistry, Soil Science and Microbiology at the University of Córdoba in Spain conducted blind sensory analyses. To this end, the official tasting sheet provided by the International Organisation of Vine and Wine (OIV) was used [30]. The attributes were limpidity, appearance, olfactory genuineness, olfactory intensity, olfactory quality, taste genuineness, taste intensity, taste persistence, taste quality, and harmony/overall judgement. All samples were refrigerated at 10 °C for 24 h prior to analysis. Each judge was offered 30 mL of wine per sample, served at 10 °C in standardised tasting glasses in accordance with the NF V09-110 AFNOR (1995) standard and the requirements of ISO 3591. The wines were presented in a random order and identified by three-digit codes. One-minute intervals were maintained between each sample.

### 2.5. Statistical Analysis

A statistical analysis was performed using ANOVA to determine whether the observed differences were significant. When significant differences were identified, a Tukey post hoc analysis (*p* < 0.05) was conducted to categorise them into homogeneous groups. All analyses were performed using IBM SPSS Statistics 25 (Armonk, New York, NY, USA).

Furthermore, heat maps and cluster analyses were conducted using open-source Python 3.9.7 code within the Anaconda Jupyter Project environment (Anaconda Inc., Austin, TX, USA).

## 3. Results and Discussion

### 3.1. Oenological Parameters

The general parameters of the wines are shown in Table 1. A reduction in pH and an increase in titratable acidity are observed as a result of lactic acid production by *L. thermotolerans*. Specifically, the reduction in pH is between 0.14 and 0.17 points, which is consistent with the findings of other studies that reported reductions between 0.12 and 0.24 points [8,23]. Vaquero et al. [8] tested sequential inoculations of *L. thermotolerans* and co-inoculations of *L. thermotolerans* with other non-*Saccharomyces* yeasts. They found pH reductions in wines produced with *L. thermotolerans* and in those obtained with *L. thermotolerans* and *M. pulcherrima*. Co-inoculations with other non-*Saccharomyces* yeasts of the *Hanseniaspora* and *Torulaspora* genera inhibited this pH reduction, probably due to the lower implantation of *L. thermotolerans*, which is less competitive than previous yeasts.

Titratable acidity increases in wines due to lactic acid production. However, differences were observed when the yeasts were inoculated in different orders (LT and LT+SC or MP+LT), with less production in the second case. This reduction may be attributed to a lower implantation rate of L. *thermotolerans*, or to nitrogen depletion in the must caused by early consumption by *M. pulcherrima*. This is because the nitrogen and amino acid content of the must, and the production of lactic acid by this yeast, are closely related [31].

The presence of *L. thermotolerans* reduces ethanol in wines by 0.4% *v/v*, regardless of the fermentation conditions. This is due to this yeast having a lower sugar-to-ethanol yield than *S. cerevisiae*, which favours respiratory metabolism and the production of lactic acid from sugars, as a result of a weakened Crabtree effect [9]. In the case of sequential fermentation involving *L. thermotolerans* and *S. cerevisiae*, the reduction in ethanol content is comparable to that observed when *L. thermotolerans* is inoculated alone. This may be because most of *L. thermotolerans*’ differential metabolism occurs at the beginning of fermentation, as evidenced by the amount of lactic acid produced, and as verified by other authors [7]. In the case of *M. pulcherrima* + *L. thermotolerans*, however, no decrease in ethanol content is observed. This may be due to the lower implantation of *L. thermotolerans*, as evidenced by the lower levels of lactic acid, which may be the result of greater competition between the two yeasts or a lower nutrient content. This is because *L. thermotolerans* produces the most lactic acid in the first few days of fermentation, a process that is highly dependent on the nutrients available in the must. Other authors have also not found decreases in ethanol content in *M. pulcherrima* inoculations alone [32].

Another noteworthy aspect is that *L. thermotolerans* produces higher levels of volatile acidity than the control wine. This is because one of the pathways used to synthesise lactic acid is similar to that used for producing acetic acid [33]. In this process, lactic acid is formed from pyruvic acid, and reducing power in the form of NADH is consumed. The inability to convert pyruvic acid to ethanol, which would normally reoxidise coenzymes, suggests that wines produced with Lachancea thermotolerans have a lower ethanol content. This phenomenon is comparable to glycero-pyruvic fermentation. To restore the redox balance, compounds such as acetic acid can be generated from acetaldehyde, thereby restoring the NADH/NAD^+^ balance.

### 3.2. Volatile Aroma Compounds

**Figure 2:**
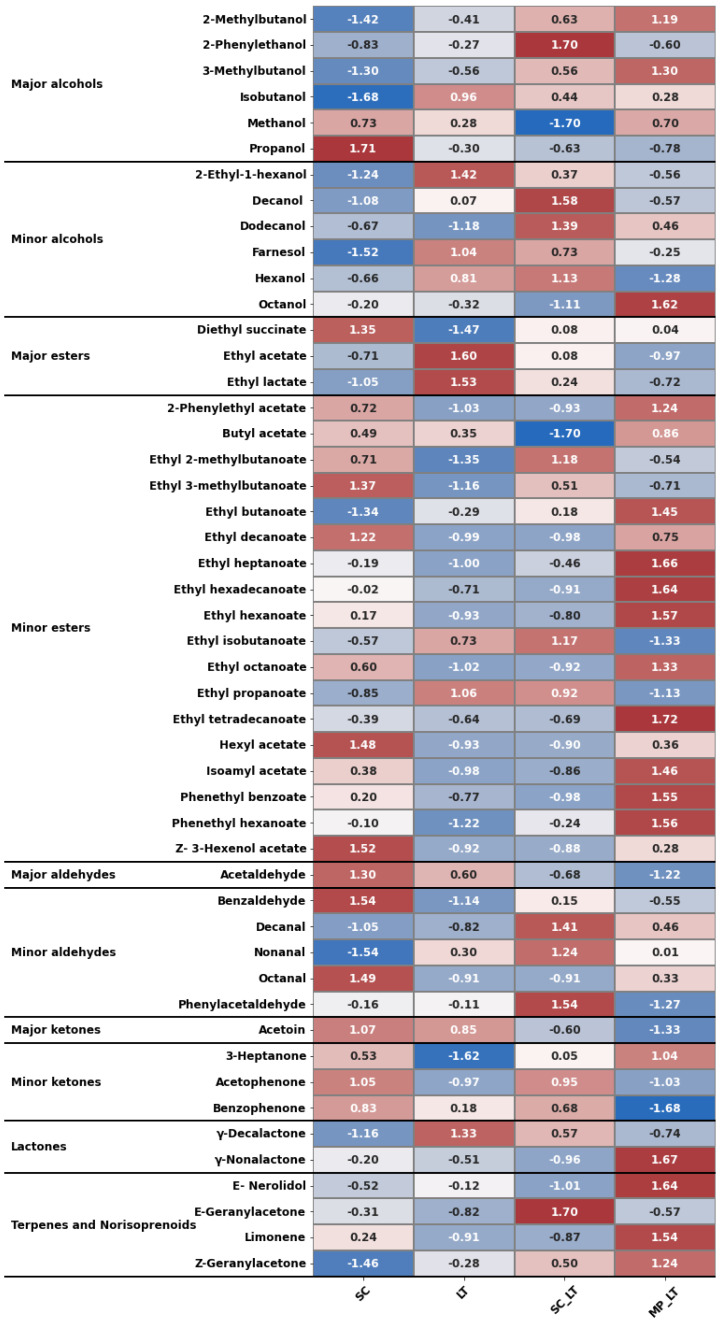
Heatmap of z-normalised data for volatile compounds. SC: Pure culture of *S. cerevisiae* (control); LT: Pure culture of *L. thermotolerans*; SC_LT: *L. thermotolerans* followed by *S. cerevisiae* after 48 h; MP_LT: *M. pulcherrima* followed by *L. thermotolerans* after 48 h. Values above 0 indicate a higher-than-average concentration (red), while values below 0 (blue) indicate a lower-than-average concentration.

Table 2 shows the concentrations of the volatile aroma compounds determined in each wine. To identify the compounds that deviate from the average, a heat map (Figure 2) was created, where values close to 0 indicate a concentration close to the average. Values above 0 indicate a higher-than-average concentration (red), while values below 0 (blue) indicate a lower-than-average concentration.

In the case of both major and minor alcohols, wines fermented only with *S. cerevisiae* generally have lower values. Conversely, wines obtained using a combination of *L. thermotolerans* and *S. cerevisiae* have the highest values. Notably, 2-phenylethanol, decanol, and dodecanol stand out among these. However, this is less evident in the total amount of higher alcohols due to *S. cerevisiae* producing more propanol, although the opposite is true for the other higher alcohols.

In the case of esters, wines produced using *L. thermotolerans* have lower values, except for ethyl acetate and ethyl lactate. These two esters are related to the acetic and lactic acid content, which is higher in these wines (see Table 1). Wines produced using *M. pulcherrima* generally have higher ester levels, particularly of ethyl hexanoate, heptanoate, tetradecanoate, hexadecanoate, and 2-phenylethanol benzoate. As esters contribute fruity notes to the aroma of wine [34], higher levels of these esters are expected to contribute positively to the aromatic quality of the wine. In this sense, the combination of *M. pulcherrima* and *L. thermotolerans* compensates for the low ester production when wine is produced solely with *L. thermotolerans*, as demonstrated in numerous studies [21,35], whose wines contain a third of the esters of the control. Recent studies have highlighted that *M. pulcherrima* produces notable levels of fruity and floral esters, including ethyl acetate, isoamyl acetate, and phenylethyl acetate [36,37]. Furthermore, in ice wine fermentations, the early addition of *M. pulcherrima* increased the production of ethyl caprylate, ethyl hexanoate, and ethyl heptanoate [36]. These compounds contribute aromas reminiscent of banana, pear, rose, and tropical fruits. Therefore, the combination of *L. thermotolerans* and *M. pulcherrima* is a promising approach to enhancing the acidity and sensory quality of wines produced solely with *L. thermotolerans*. Other authors have also demonstrated that combining both yeasts increases ester production compared to the control, particularly 2-phenylethanol acetate [8].

The content of carbonyl compounds (aldehydes and ketones) is higher in control wine, particularly acetaldehyde, benzaldehyde, and octanal. The lowest levels are found in wines produced using *L. thermotolerans*.

Regarding lactones, *L. thermotolerans* stands out for its high production of γ-decalactone. It is believed that this lactone can be produced during fermentation through the cyclisation of 10-hydroxypalmitic acid, a by-product of palmitoleic acid [38]. This suggests that the metabolic pathway of *L. thermotolerans* is more active than that of *S. cerevisiae*, as some authors have previously suggested [39].

Terpene compounds originate mainly in grapes and have floral notes. Most of them are bound to a carbohydrate residue and are therefore not volatile [40]. The cleavage and subsequent release of terpenes in their odorous forms depends on various factors, including the amount present in the must, the yeast’s enzymatic activity, and the pH [34]. Their behaviour is like that of esters, as wines produced solely with *L. thermotolerans* have lower content than those produced with a combination of *M. pulcherrima* and *L. thermotolerans*. This is probably due to the latter having lower glycosidase activity [10]. Other authors have demonstrated that using *M. pulcherrima* alongside other yeasts increases the concentration of terpenoids compounds, establishing it as a biotechnological tool for enhancing the aromatic profile of wine [10,41].

### 3.3. Odor Activity Values and Aromatic Series

Odour activity values (OAVs) are defined as the ratio of a volatile compound’s concentration to its sensory perception threshold (Table S2). They are an essential tool for evaluating the sensory relevance of aroma-active compounds. Compounds with OAVs greater than one are considered to actively contribute to the aroma of the wine, while those below this threshold may still exert synergistic or background effects [42]. The 13 volatile aroma compounds isoamyl acetate, Z-3-hexenol acetate, 2-phenylethyl acetate, ethyl propanoate, ethyl butanoate, ethyl 3-methylbutanoate, ethyl isobutanoate, ethyl hexanoate, ethyl octanoate, ethyl decanoate, octanal, nonanal, and decanal, as well as the non-volatile compound phenylacetaldehyde, all exhibit OAVs greater than one. Together, these compounds account for more than 90% of the total OAV for a given wine. Many of these compounds have also been described by other authors as being primarily responsible for the aroma of wine [43,44]. Most of these compounds are ethyl esters with fruity aromas, and wines produced with *M. pulcherrima* and *L. thermotolerans* show the highest OAV values. The values of octanal are also highlighted in wines produced with only *S. cerevisiae*, as well as in those obtained with *M. pulcherrima* and *L. thermotolerans*.

**Figure 3:**
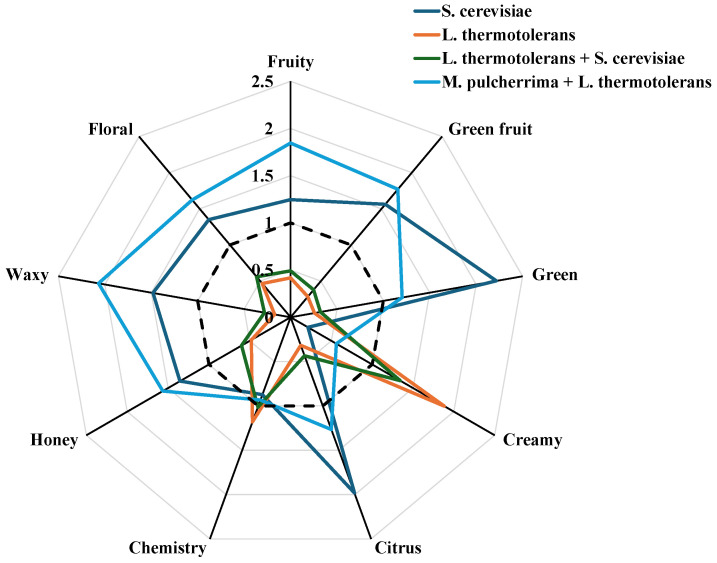
Star plot, obtained by multivariate data analysis of aroma compounds grouped in aroma series, of the produced wines.

Identifying individual odorants is important, but grouping volatile compounds into aroma series provides a higher-level framework for interpreting the sensory profile of wines. This approach aggregates compounds with similar aromatic descriptors, enabling a more comprehensive description of aroma complexity [29,44,45,46,47,48]. Together, the use of OAVs and aroma series enables both the quantitative and qualitative interpretation of wine aroma, thereby enhancing our understanding of how chemical composition translates into sensory perception. Nine aromatic series were identified and selected to create a fingerprint of the wines (Table 3 and Figure 3): fruity, green fruit, green, creamy, citrus, chemical, honey, waxy, and floral. The fruity series contributes the most to the aroma of all wines. The green fruit, floral, honey, citrus, and waxy series show the highest values in wines produced with only *S. cerevisiae*, as well as in those produced with *M. pulcherrima* and *L. thermotolerans*.

By contrast, wines that are initially fermented with *L. thermotolerans* stand out in the creamy series because this yeast produces higher concentrations of the main compounds that contribute to this aromatic category—lactones, acetoin, and ethyl lactate—due to its specific metabolic pathways [7,21,49].

### 3.4. Multivariate Analysis, Cluster Analysis, and Heat Map

A multivariate analysis was used to construct a star plot with nine axes, each representing a different aroma series (see Figure 3). Prior to plotting, the data for each series was standardised to ensure that all rays had the same maximum length, with a unit value corresponding to the average for each series. This standardisation enables meaningful comparisons to be made across the series. This visualisation makes it possible to identify which aroma series are dominant in a given sample and detect similarities among observations, thereby facilitating the identification of potential clusters [50]. As can be seen, the use of different yeasts gives rise to wines with different fingerprints, although the control wine and those produced with *M. pulcherrima* and *L. thermotolerans* show a similar pattern. In any case, except for the green, citrus, and chemistry series, the latter shows higher values in the aromatic series. Wines produced with *L. thermotolerans* alone or with *S. cerevisiae* show quite similar behaviour, highlighting only the creamy series and the chemistry series to a slight extent.

Cluster analysis is an exploratory technique that classifies objects or cases into groups (or ‘clusters’) based on their similarities. To achieve this, a set of variables is selected as the classification factors. The closer two clusters are, the more similar the samples that make up both clusters are [51]. In this case, it has been combined with a heat map of normalised scores to make the result and the effect of each variable easier to understand.

**Figure 4:**
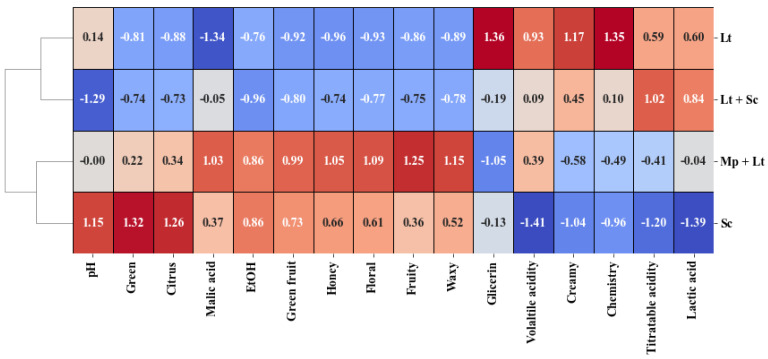
Cluster analysis and heatmap of the wines based on oenological variables and the aromatic series with the normalised scores. SC: Pure culture of *S. cerevisiae* (control); LT: Pure culture of *L. thermotolerans*; SC **+** LT: *L. thermotolerans* followed by *S. cerevisiae* after 48 h; MP+LT: *M. pulcherrima* followed by *L. thermotolerans* after 48 h. Values above 0 indicate a higher-than-average concentration (red), while values below 0 (blue) in-dicate a lower-than-average concentration.

The general parameters and aromatic series were used as classification variables. As can be seen in Figure 4, there are two distinct groups: the control and MP+LT wines on the one hand, and those fermented with *L. thermotolerans* on the other. The latter are positively correlated with most oenological parameters, including volatile acidity, titratable acidity, lactic acid, and glycerol, as well as the creamy and chemical series. The remaining parameters and series are positively correlated for wines produced using *S. cerevisiae* or *M. pulcherrima* and *L. thermotolerans*. Wines obtained using *M. pulcherrima* and *L. thermotolerans* show intermediate values for variables such as pH and lactic acid between the control and those obtained with *L. thermotolerans* alone or together with *S. cerevisiae*.

Therefore, we can conclude that using *L. thermotolerans* positively influences oenological variables, whereas combining *M. pulcherrima* and *L. thermotolerans* significantly affects the aromatic composition.

### 3.5. Organoleptic Characterisation

**Figure 5:**
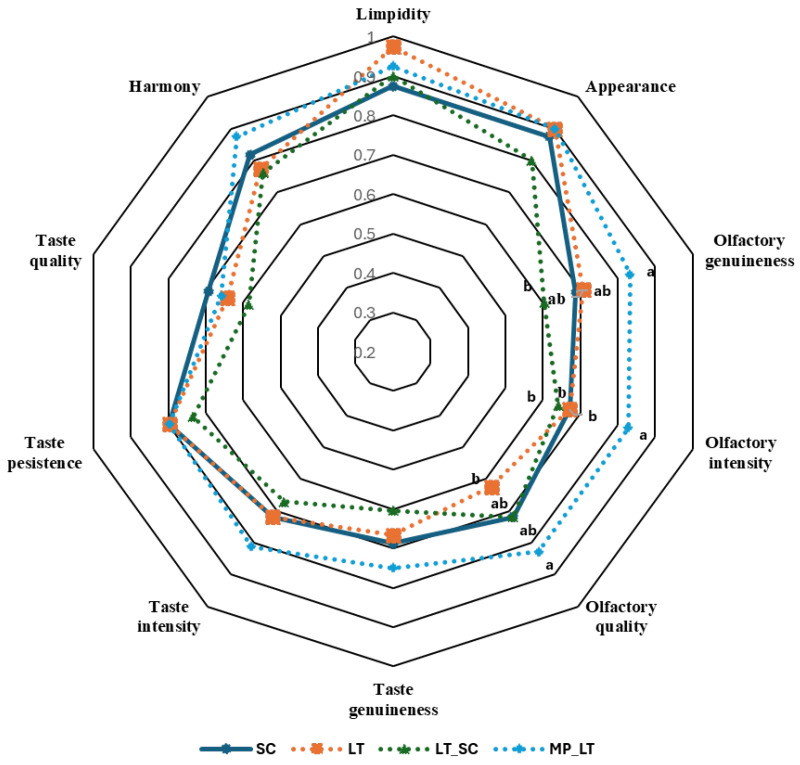
Starplot of the value obtained by each wine against the maximum possible score in each section of the tasting. Different letters indicate significant differences at 95% confidence level.

An organoleptic characterisation was carried out using the OIV tasting sheet described in Section 2.4, with the aim of determining the effect of the yeasts used on the quality of the wines as perceived by a tasting panel. To this end, the ten parameters listed on the tasting sheet were evaluated. The data obtained (Figure 5) were represented by plotting the value obtained by each wine against the maximum possible score in each section of the tasting.

The wines made with the combination of *M. pulcherrima* and *L. thermotolerans* stood out from the rest in the organoleptic characterisation.

These wines performed particularly well in the olfactory phase, achieving higher scores than the others in terms of intensity, purity, and olfactory quality—even surpassing the control wine. In these same aspects, wines fermented by *L. thermotolerans* performed poorly.

In terms of appearance, wines fermented by *L. thermotolerans* alone stand out, probably due to greater brightness and clarity resulting from higher acidity.

In the taste section, no statistically significant differences were observed, although the wine fermented with *M. pulcherrima* stood out in terms of taste intensity, probably due to its superior aroma reaching the tasters via the retronasal passage.

Finally, in terms of aroma, referring to the overall sensation of the wine, the wine fermented with *M. pulcherrima* and *L. thermotolerans* stands out above the control wine, while the wine fermented with *L. thermotolerans* alone stands out above the others.

In general, the organoleptic characterisation is consistent with the other sections of the article, confirming that combining both yeasts produces wines with greater aromatic and gustatory complexity when starting with a must that is low in acidity and has a neutral aroma, such as that of Pedro Ximénez grapes. This is similar to the findings of other authors regarding varieties such as Airén, which is the most widely planted variety in Spain [15].

## 4. Conclusions

The combination of *Lachancea thermotolerans* and *Metschnikowia pulcherrima* has shown a clear synergistic effect in improving the oenological and sensory properties of Pedro Ximénez white wines from warm climates. *L. thermotolerans* significantly increased titratable acidity (up to 6.83 g/L), reduced pH (down to 3.02), and raised lactic acid levels (up to 2.0 g/L), enhancing the freshness and microbial stability of the wine. It also produced higher levels of γ-decalactone and ethyl lactate, contributing creamy notes. Meanwhile, *M. pulcherrima* contributed to a notable rise in esters such as ethyl hexanoate, ethyl octanoate, and 2-phenylethyl acetate, which are responsible for fruity, floral, and honey aromas. Wines that were fermented sequentially with *M. pulcherrima* and *L. thermotolerans* had the highest values for total olfactory activity, particularly in the fruity and floral aromatic categories. These values even surpassed those of the control wines (*S. cerevisiae*). However, *L. thermotolerans* produced lactic acid at a lower rate, probably due to greater competition between the two yeasts. Sensory analysis confirmed these results, with these wines scoring highest in olfactory intensity, aroma quality, and overall harmony. Cluster and multivariate analyses also revealed that this yeast combination led to a distinct aromatic fingerprint, supporting its use as an effective biotechnological strategy to enhance wine quality under climate change conditions, especially in neutral or low-acidity musts.

## Figures and Tables

**Table 1:** General oenological parameters determined in the wines.

		SC	LT	LT → SC	MP → LT
pH		3.29 ± 0.01 a	3.15 ± 0.01 b	3.02 ± 0.04 c	3.18± 0.01 b
Titratable Acidity	g/L TH_2_	4.56 ± 0.04 d	6.38 ± 0.05 b	6.83 ± 0.05 a	5.4 ± 0.1 c
Ethanol	% *v/v*	13.47 ± 0.06 a	13.07 ± 0.06 b	13.02 ± 0.03 b	13.47 ± 0.06 a
Volatile Acidity	g/L AcH	0.35 ± 0.02 c	0.61 ± 0.02 a	0.52 ± 0.02 b	0.55 ± 0.02 b
Lactic Acid	g/L	N.D.	1.8 ± 0.1 a	2.0 ± 0.1 a	1.24 ± 0.06 b
Malic Acid	g/L	0.61 ± 0.06 ab	0.55 ± 0.03 b	0.55 ± 0.06 b	0.71 ± 0.06 a
Glycerol	g/L	4.41 ± 0.16 ab	4.82 ± 0.36 a	4.39 ± 0.23 ab	4.15 ± 0.05 c

TH_2_: Tartaric acid; AcH: Acetic acid; SC: Pure culture of *S. cerevisiae* (control); LT: Pure culture of *L. thermotolerans*; SC **→** LT: *L. thermotolerans* followed by *S. cerevisiae* after 48 h; MP **→** LT: *M. pulcherrima* followed by *L. thermotolerans* after 48 h. N.D.: Not detected. Different letters indicate significant differences at 95% confidence level.

**Table 2:** Volatile compounds determined in wines.

	SC	LT	LT → SC	MP → LT
**Alcohols**				
**Major alcohols (mg/L)**	**503.7 ± 0.3 a**	**469 ± 2 a**	**461 ± 3 a**	**470.9 ± 0.9 a**
Methanol	50 ± 1 a	47.98 ± 0.09 a	40 ± 9 a	50 ± 2 a
Propanol	149.8 ± 0.6 a	61.8 ± 0.7 b	47 ± 9 c	40.62 ± 0.09 c
Isobutanol	26.21 ± 0.09 c	66 ± 1 a	58 ± 4 b	55.7 ± 0.6 b
2-methylbutanol	27.5 ± 0.2 d	34.2 ± 0.2 c	41 ± 2 b	44.9 ± 0.3 a
3-methylbutanol	227.42 ± 0.09 d	236 ± 4 c	248 ± 2 b	257 ± 2 a
2-phenylethanol	23 ± 0.5 a	23.5 ± 0.3 a	25 ± 2 a	23.2 ± 0.2 a
**Minor alcohols (µg/L)**	**1022 ± 79 b**	**1562 ± 57 a**	**1666 ± 105 a**	**837 ± 88 b**
Hexanol	964 ± 82 b	1499 ± 57 a	1616 ± 103 a	739 ± 85 c
2-ethyl-1-hexanol	22 ± 2	24 ± 1	23.1 ± 0.9	22 ± 2
Octanol	29 ± 4 b	26 ± 2 b	10 ± 1 c	65 ± 4 a
Decanol	5 ± 2 a	6 ± 0.4 a	7.6 ± 0.8 a	5.3 ± 0.7 a
Dodecanol	1.2 ± 0.1 c	0.84 ± 0.08 d	2.7 ± 0.2 a	2 ± 0.06 b
Farnesol	1.7 ± 0.3 c	6.6 ± 0.6 a	6 ± 0.4 a	4.2 ± 0.5 b
**Esters**				
**Major esters (mg/L)**	**97.1 ± 0.4** a	**167.6 ± 0.8** a	**128 ± 10** a	**94 ± 0.7** a
Ethyl acetate	70 ± 1 b	118 ± 2 a	87 ± 24 ab	65 ± 1 b
Ethyl lactate	18.24 ± 0.09 d	42.4 ± 0.9 a	34 ± 5 b	21.3 ± 0.09 c
Diethyl succinate	8.7 ± 0.3 a	6.99 ± 0.05 b	7.91 ± 0.06 c	7.89 ± 0.01 b
**Minor esters (µg/L)**	**9289 ± 629 b**	**2660 ± 155 c**	**3126 ± 54 c**	**12,497 ± 394 a**
Ethyl propanoate	66 ± 7 b	121 ± 10 a	117.2 ± 0.9 a	58 ± 3 b
Ethyl isobutanoate	30 ± 1 c	62 ± 7 b	73 ± 2 a	10.8 ± 0.3 d
Ethyl butanoate	142 ± 8 c	203 ± 17 b	230 ± 6 b	303 ± 9 a
Butyl acetate	0.33 ± 0.04 a	0.32 ± 0.04 a	N.D.	0.4 ± 0.1 a
Ethyl 2-methylbutanoate	3 ± 0.2 b	0.96 ± 0.09 d	3.5 ± 0.1 a	1.8 ± 0.2 c
Ethyl 3-methylbutanoate	7.2 ± 0.5 a	2.2 ± 0.2 b	5.5 ± 0.3 c	3.1 ± 0.2 c
Isoamyl acetate	4611 ± 327 b	1722 ± 106 c	1978 ± 38 c	6910 ± 237 a
Ethyl hexanoate	486 ± 26 b	162 ± 15 c	200 ± 5 c	894 ± 55 a
Z-3- hexenol acetate	473 ± 26 a	15 ± 1 c	21 ± 0.6 c	239 ± 11 b
Hexyl acetate	193 ± 18 a	6.4 ± 0.5 c	8.9 ± 0.8 c	106 ± 5 b
Ethyl heptanoate	0.27 ± 0.02 b	0.12 ± 0.01 c	0.22 ± 0.01 b	0.61 ± 0.04 a
Ethyl octanoate	600 ± 42 b	48 ± 5 c	82 ± 5 c	849 ± 41 a
2-phenylethanol acetate	1815 ± 192 b	159 ± 4 c	253 ± 6 c	2302 ± 82 a
Ethyl decanoate	775 ± 44 a	98 ± 4 c	100.96 ± 0.04 c	632 ± 35 b
Phenethyl hexanoate	0.42 ± 0.01 b	0.31 ± 0.01 c	0.41 ± 0.01 b	0.59 ± 0.04 a
Ethyl tetradecanoate	21 ± 2 b	15.8 ± 0.5 b	14.8 ± 0.2 b	66 ± 6 a
Phenethyl benzoate	1.11 ± 0.07 ab	1.04 ± 0.03 b	1.02 ± 0.03 b	1.21 ± 0.05 a
Ethyl hexadecanoate	65 ± 4 b	42.5 ± 0.8 c	36 ± 1 c	119 ± 11 a
**Aldehydes**				
**Major aldehydes (mg/L)**	**70 ± 2** a	**64 ± 4** a	**51 ± 3** b	**45.7 ± 0.8** b
Acetaldehyde	71 ± 2 a	64 ± 4 a	51 ± 3 b	46.1 ± 0.8 b
**Minor aldehydes (µg/L)**	**131 ± 7 a**	**27 ± 2 c**	**34 ± 3 c**	**82 ± 2 b**
Benzaldehyde	3.8 ± 0.1 a	2.5 ± 0.3 b	3.1 ± 0.3 b	2.8 ± 0.3 b
Octanal	105 ± 5 a	1.8 ± 0.2 c	2 ± 0.1 c	55 ± 2 b
Nonanal	3.4 ± 0.2 b	4.7 ± 0.5 a	5.3 ± 0.6 a	4.5 ± 0.4 ab
Decanal	5.6 ± 0.6 c	5.9 ± 0.6 bc	9 ± 1 a	7.7 ± 0.7 ab
Phenylacetaldehyde	12 ± 1 ab	12 ± 0.8 ab	14 ± 1 b	10.5 ± 0.5 a
**Ketones**				
**Major Ketones (mg/L)**	**15.2 ± 0.4 a**	**14.9 ± 0.3 a**	**12 ± 1 b**	**11.2 ± 0.5 b**
Acetoin	15.2 ± 0.4 a	14.9 ± 0.3 a	12 ± 1 b	11.2 ± 0.5 b
**Minor Ketones (µg/L)**	**13.9 ± 0.7 a**	**5.5 ± 0.3 a**	**13 ± 1 b**	**6.5 ± 0.5 b**
Benzophenone	0.54 ± 0.08 a	0.4 ± 0.02 b	0.51 ± 0.02 a	N.D.
3-Heptanone	3.9 ± 0.4 a	2.6 ± 0.2 a	3.6 ± 0.2 b	4.2 ± 0.4 a
Acetophenone	9.4 ± 0.6 a	2.48 ± 0.09 a	9 ± 1 b	2.2 ± 0.2 b
**Lactones (µg/L)**	**10 ± 1 d**	**172 ± 3 a**	**121 ± 5 b**	**43 ± 3 c**
G-Nonalactone	8 ± 1 b	7 ± 0.9 bc	5.7 ± 0.3 c	13.6 ± 0.5 a
G-Decalactone	1.6 ± 0.1 d	165 ± 3 a	115 ± 6 b	29 ± 3 c
**Terpenoids (µg/L)**	**12.6 ± 0.4 b**	**10 ± 0.4 c**	**12.4 ± 0.6 b**	**15.9 ± 0.7 a**
Limonene	7.5 ± 0.5 b	5.1 ± 0.5 c	5.1 ± 0.3 c	10.3 ± 0.7 a
E-nerolidol	2.64 ± 0.04 b	2.65 ± 0.02 ab	2.62 ± 0.02 b	2.71 ± 0.02 a
E-geranyl acetone	1 ± 0.1 b	0.57 ± 0.06 b	2.7 ± 0.3 a	0.78 ± 0.08 b
Z-geranyl acetone	1.5 ± 0.1 b	1.8 ± 0.2 ab	1.9 ± 0.2 a	2.1 ± 0.2 a

SC: Pure culture of *S. cerevisiae* (control); LT: Pure culture of *L. thermotolerans*; SC → LT: *L. thermotolerans* followed by *S. cerevisiae* after 48 h; MP → LT: *M. pulcherrima* followed by *L. thermotolerans* after 48 h. N.D.: Not detected. Different letters indicate significant differences at 95% confidence level.

**Table 3:** Aromatic series obtained in the wines.

	SC	LT	LT → SC	MP → LT
Fruity	332 ± 22 b	111 ± 7 c	131 ± 3 c	492 ± 20 a
Green fruit	74 ± 4 b	14 ± 1 c	18 ± 0 c	84 ± 5 a
Green	40 ± 2 a	5 ± 0 c	6 ± 0 c	22 ± 1 b
Creamy	1 ± 0.1 d	5 ± 0.2 a	3.3 ± 0.2 b	1.4 ± 0.1 c
Citrus	49 ± 3 a	8 ± 1 c	11 ± 1 c	31 ± 1 b
Chemistry	21 ± 1 b	29 ± 2 a	25 ± 3 ab	23 ± 1 ab
Honey	10 ± 1 b	4 ± 0 c	4.5 ± 0.3 c	12 ± 0 a
Waxy	128 ± 9 b	15 ± 1 c	24 ± 2 c	179 ± 8 a
Floral	10 ± 1 b	3 ± 0.2 c	4 ± 0.2 c	12.8 ± 0.9 a

SC: Pure culture of *S. cerevisiae* (control); LT: Pure culture of *L. thermotolerans*; SC **→** LT: *L. thermotolerans* followed by *S. cerevisiae* after 48 h; MP **→** LT: *M. pulcherrima* followed by *L. thermotolerans* after 48 h. Different letters indicate significant differences at 95% confidence level.

## Data Availability

The original contributions presented in this study are included in the article/Supplementary Materials. Further inquiries can be directed to the corresponding author.

## Supplementary Materials

The following supporting information can be downloaded at: https://www.mdpi.com/article/10.3390/microorganisms13081908/s1, Table S1: Major and minor volatile aroma compounds identified in the wines. Table S2: Odor descriptor, odor threshold and aroma series assigned to the volatile compounds identified in the wines analyzed.

## Abbreviations

The following abbreviations are used in this manuscript:

GC-FID	Gas chromatography flame ionisation detector
GC-MS	Gas chromatography mass spectrum detector
PX	Pedro Ximenez grape variety
