# Supplementary material for: Climate Change Adaptation in Winemaking: Combined Use of Non-Saccharomyces Yeasts to Improve the Quality of Pedro Ximénez Wines
Source: Microorganisms. 2025 Aug 15;13(8):1908. doi: 10.3390/microorganisms13081908 (PMC12388478; doi:10.3390/microorganisms13081908)
Supplement: Supplementary file 1 [file microorganisms-13-01908-s001.zip › microorganisms-3777820-supplementary.pdf]

Table S1. *Major and minor volatile aroma compounds identified in the wines.*

Compound	Prv‡	CAS[c]	LRI[a]	LRI[b]	Slope	Interception	R^2	LOD[d] (µg/L)	LOQ[e] (µg/L)
**Alcohols**									
**Mayor Alcohols**									
Methanol*	M	67-56-1	925	879	5.04E-03	6.0E-03	0.995	1,1E+04	3,8E+04
Propanol*	S	71-23-8	1068	1060	9.1E-03	5.0E-03	0.995	1,3E+04	4,4E+04
Isobutanol*	S	78-83-1	1126	1108	9.5E-03	3.0E-03	0.999	1,4E+04	4,7E+04
Isoamyl alcohols†*	M	123-51-3	1243	1230	8.7E+03	9.0E-03	0.999	1,0E+05	3,3E+05
2-phenylethanol*	S	60-12-8	2011	1892	1,10E-02	0,002	0.999	1,3E+04	4,2E+04
**Minor Alcohols**									
Hexanol	F	111-27-3	867	867	1.00E-03	0.00E+00	0.944	1,1E+03	3,6E+03
2-ethyl-1-hexanol	S	104-76-7	1028	1027	1.04E-03	8.30E-06	0.955	8,7E+00	2,9E+01
Octanol		111-87-5	1060	1064	8,51E-05	7,24E-03	0,949	6,2E+00	2,1E+01
Decanol	M	123-51-3	1243	1230	8.7E+03	9.0E-03	0.999	2,6E+00	8,7E+00
Dodecanol	S	112-53-8	1461	1469	2.60E-03	8.65E-02	0.982	1,6E-01	5,2E-01
Farnesol		4602-84-0	1728	1740	6.65E-003	2.97E-01	0.992	2,1E+00	6,9E+00
**Esters**									
**Mayor Esters**									
Ethyl acetate*	S	141-78-6	906	885	4.96E-03	-3.0E-04	0.998	1,5E+04	5,1E+04
Ethyl lactate*	S	97-64-3	1354	1326	5.7E-03	0,03	0.999	3,6E+04	1,2E+05
Diethyl succinate*	S	123-25-1	1730	1702	5.8E-03	2.8E-02	0.996	4,7E+03	1,6E+04
**Minor Esters**									

Compound	Prv‡	CAS[c]	LRI[a]	LRI[b]	Slope	Interception	R^2	LOD[d] (μg/L)	LOQ[e] (μg/L)
Ethyl propanoate		105-37-3	714	715	1.04E-03	0.00	0.985	1,6E+01	5.2E+01
Ethyl isobutanoate	S	97-62-1	753	755	8.22E-02	1.64E-02	0.989	2,9E+01	9,6E+01
Ethyl butanoate	S	105-54-4	802	802	7.80E-03	1.03E-03	0.989	1,2E+01	4,1E+01
Butyl acetate		123-86-4	823	819	7,06E-03	3,34E-02	0,978	9,0E-01	3,0E+00
Ethyl 2-methylbutanoate		7452-79-1	847.4	846	1,66E-03	4,19E-03	0,987	1,2E+00	4,0E+00
Ethyl 3-methylbutanoate	S	108-64-5	851	847	7.78E-03	1.03E-02	0.989	2,0E+00	6,6E+00
Isoamyl acetate	F	123-92-2	875	876	4.80E-02	1.12E-02	0.984	1,4E+02	4,5E+02
Ethyl hexanoate	F	123-66-0	1000	1001	7.02E-02	4.32E-02	0.917	2,6E+01	8,7E+01
Z-3 Hexenyl acetate		3681-71-8	1007	1005	2.47E-03	1.20E-02	0.984	4.1E+00	1.3E+01
Hexyl acetate	S	142-92-7	1014	1015	1.34E-01	1.65E-02	0.991	3,6E+00	1,2E+01
Ethyl heptanoate		106-30-9	1102	1095	3,69E-02	5,71E-03	0,991	1,3E-01	4,3E-01
Ethyl octanoate	F	106-32-1	1198	1196	1.78E-01	-4.70E-03	0.988	1,4E+01	4,5E+01
2-phenylethyl acetate	S	103-45-7	1256	1256	6.22E-02	4.10E-03	0.995	9,4E+01	3,1E+02
Ethyl decanoate	F	110-38-3	1395	1397	1.69E-01	6.90E-03	0.976	1,1E+01	3,6E+01
Phenethyl hexanoate		6290-37-5	1654	1646	5,31E-02	-2,34E-02	0,992	6,2E-02	2,1E-01
Ethyl tetradecanoate	F	124-06-1	1793	1793	1.84E-01	6.51E-03	0.987	1,7E+00	5,6E+00
Phenethyl benzoate		94-47-3	1859	1859	4,06E-02	-3,15E-02	0,985	2,9E-01	9,8E-01
Ethyl hexadecanoate	F	628-97-7	1992	1996	1.83E-01	-2.60E-03	0.971	5,8E+00	1,9E+01
**Aldehydes**									
**Mayor Aldehydes**									
Acetaldehyde*	S	75-07-0	688	800	4.23E-03	-7.0E-04	0.999	1,7E+04	5,5E+04
**Minor Aldehydes**									
Benzaldehyde	S	100-52-7	958.9	959	5.10E-03	7.00E-03	0.988	3,2E-01	1,1E+00

Compound	Prv‡	CAS[c]	LRI[a]	LRI[b]	Slope	Interception	R^2	LOD[d] (µg/L)	LOQ[e] (µg/L)
Heptanal	S	111-71-7	903	901	7.90E-03	2.56E-03	0.961	1,2E-01	3,9E-01
Octanal	S	124-13-0	1004	1004	9.10E-03	1.80E-02	0.961	3,2E-01	1,1E+00
Nonanal	S	124-19-6	1104	1103	9.90E-03	1.79E-02	0.979	1,4E-01	4,5E-01
Decanal	S	112-31-2	1206	1206	8.90E-03	1.78E-02	0.959	2,9E-01	9,5E-01
Phenylacetaldehyde		122-78-1	1046	1050	6,25E-04	3,56E-03	0,965	5,3E+00	1,8E+01
**Ketones**									
**Mayor ketones**									
Acetoin*	S	513-86-0	1337	1309	5.08E-03	-0,001	0.997	1,7E+04	5,7E+04
**Minor ketones**									
Benzophenone		119-61-9	1626	1625	1,27E-02	9,19E-02	0,993	1,5E-01	5,0E-01
3-Heptanone	S	106-35-4	1130	1141	3.28E-003	2.88E-02	0.910	3,8E-02	1,3E-01
Acetophenone		98-86-2	1065	1066	1.35E-03	1.33E-02	0.959	5.9E-01	1.9E+00
**Lactones**									
γ-Nonalactone	M	104-61-0	1363	1362	4.90E-03	-1.00E-04	0.943	6,6E+00	2,2E+01
γ-Decalactone	S	706-14-9	1470	1470	7.34E-02	-1.31E-02	0.983	5,0E+00	1,7E+01
**Terpenes & Norisoprenoids**									
Limonene	S	138-86-3	1022	1030	3.30E-02	2.70E-03	0.976	8,5E+00	2,8E+01
E-Nerolidol		142-50-7	1551	1550	1.41E-02	-8.87E-02	0.988	5.4E-01	1.8E+00
E-Geranyl acetone	S	689-67-8	1442	1450	5.64E-03	6.32E-03	0.976	1,6E-01	5,5E-01
Z-Geranyl acetone	S	689-67-8	1442	1450	5.64E-03	6.32E-03	0.976	5,3E-01	1,8E+00

LRI: Linear retention index using definition of Van den Dool and Kratz (1963) in a HP-5MS capillary column (30 m/0.25 mm/0.25 µm. He) and *CPWAX57-CB capillary column (60 m/0.25 mm/0.40 µm. He). . aCalculated values bData collected from the NIST Webbook of Chemistry. http://webbook.nist.gov/chemistry. cCAS: Chemical Abstracts Service

number. d LOD: Limit of Detection. e LOQ: Limit of Quantification. † Isoamyl alcohols = 2-methylbutanol + 3-methylbutanol.‡Prv: Standard providers. F: Fluka; M: Merck; S: Sigma-Aldrich.

Table S2. *Odor descriptor, odor threshold and aroma series assigned to the volatile compounds identified in the wines analyzed.*

**Compound**	**OPT**	**Descriptors**	**Serie**	**Reference**
**Alcohols**				
**Mayor Alcohols**				
Methanol	668000	Chemical, medicinal, fruity	5	(Ogawa et al., 2022)
Propanol	830000	Ripe fruit, fusel alcohol	5	(Ogawa et al., 2022)
Isobutanol	40000	Nail polish, bitter	5	(Ogawa et al., 2022)
Isoamyl alcohols	30000	Burnt, alcohol	5	(Ogawa et al., 2022)
2-phenylethanol	10000	Rose, honey, lilac	9	(Ogawa et al., 2022)
**Minor Alcohols**				
Hexanol	8000	Green, grass, oily	3	(López de Lerma et al., 2018)
2-ethyl-1-hexanol	8000	Citrus, fresh	5	(Zhang et al., 2019)
Octanol	800	Waxy, green, citrus, aldehydic and floral	8	(Peinado et al., 2006)
Decanol	400	aldehydic waxy green	8	(Qu et al., 2024)
Dodecanol	1000	waxy, earthy, soapy, aldehydic	8	(Li et al., 2008)
Farnesol	20	floral juicy	9	(Muñoz-Castells et al., 2024)
**Esters**				
**Mayor Esters**				
Ethyl acetate	7500	Fruity, glue	5	(Guth, 1997)
Ethyl lactate	150000	Fruity, byttery	4	(Peinado et al., 2004)
Diethyl succinate	100000	Overripe melon	1	(Ogawa et al., 2022)

Compound	OPT	Descriptors	Serie	Reference
**Minor Esters**				
Ethyl propanoate	10	Fruity	1	(Leffingwell & Associates, n.d.)
Ethyl isobutanoate	15	Sweet, etherial and fruity	1	(López de Lerma et al., 2018)
Ethyl butanoate	20	Fruity, floral	1	(Guth, 1997)
Butyl acetate	66	Sweet, ripe, banana, ethereal	1,6	(Takeoka et al., 1996)
Ethyl 2-methylbutanoate	18	sharp sweet green apple fruity	1,2	(Ferreira et al., 2000)
Ethyl 3-methylbutanoate	3	Pinneaple	1,2	(Ferreira et al., 2000)
Isoamyl acetate	30	sweet fruity banana	1	(Guth, 1997)
Ethyl hexanoate	14	sweet fruity pineapple green banana	1,2	(Ferreira et al., 2000)
Z-Hexenyl acetate	13	Fresh, green, apple	2,3	
Ethyl heptanoate	2,2	fruity pineapple	1	(López de Lerma et al., 2018)
Ethyl octanoate	5	Banana, pineapple	1,8	(López de Lerma et al., 2018)
2-Phenylethyl acetate	250	floral-rose and gardenia	7,9	(Guth, 1997)
Ethyl decanoate	200	Sweet, fruity, caramel, grape	1,8	(Gómez-Míguez et al., 2007)
Phenethyl hexanoate	250	Fruity-green, fresh pineapple-like	8,9	*
Ethyl tetradecanoate	4000	Tropical fruit	8	(Moreno, 2005)
Phenethyl benzoate	250	Rose, honey, balsamic	2,9	*
Ethyl hexadecanoate	2000	Waxy, fruity nuances	8	(Moreno, 2005)
**Aldehydes**				
**Mayor Aldehydes**				
Acetaldehyde	110000	Pungent, ripe apple	1,5	(Moreno, 2005)
**Minor Aldehydes**				
Benzaldehyde	350	Bitter almond, smoked	1	(Buttery et al., 1988)

Compound	OPT	Descriptors	Serie	Reference
Octanal	2,5	Citrus, green, fresh, waxy	5	(López de Lerma et al., 2018)
Nonanal	2,5	Citrus	5	(López de Lerma et al., 2018)
Decanal	1,25	Citrus	5,8	(López de Lerma et al., 2018)
Phenylacetaldehyde	4	green sweet honey	3,7	(Buttery et al., 1971)
**Ketones**				
**Mayor ketones**				
Acetoin	150000	Yogurt, butterscotch	4	(Zhang et al., 2015)
**Minor ketones**				
Benzophenone	65	Balsamic, rose, herbal, geraniun	9	(Leffingwell & Associates, n.d.)
3-Heptanone	7,5	Green, ketonic	3	(Burdock and Fenaroli, 2010)
Acetophenone	65	Mimosa, Acacia	9	(Leffingwell & Associates, n.d.)
**Lactones**				
γ -Nonalactone	30	Coconut, creamy	1,4	(Ferreira et al., 2000)
γ -Decalactone	77.7	Peach, milky	1,4	(Gottmann et al., 2023)
**Terpenes & Norisoprenoids**				
Limonene	10	citrus orange fresh sweet	5,6	(López de Lerma et al., 2018)
E-Nerolidol	700	Floral, green	3,9	(Martín-García et al., 2023)
E-Geranyl acetone	60	Magnolia, rose	9	(Zhu et al., 2019)
Z-Geranyl acetone	60	Magnolia, rose	9	(Zhu et al., 2019)

1 Fruity; 2 Green Fruit; 3 Green; 4 Creamy; 5 Citrus; 6 Chemistry; 7 Honey; 8 Waxy; 9 Floral. *Expressed as 2-phenethyl acetate; ** Expressed as Benzaldehyde; *** Expressed as Ethyl decanoate.

References

Burdock, G.A., Fenaroli, G., 2010. Fenaroli's handbook of flavor ingredients., 6th ed. Boca Raton, FL.

Buttery, R.G., Seifert, R.M., Guadagni, D.G., Ling, L.C., 1971. Characterization of additional volatile components of tomato. J Agric Food Chem 19, 524–529. https://doi.org/10.1021/jf60175a011

Buttery, R.G., Turnbaugh, J.G., Ling, L.C., 1988. Contribution of volatiles to rice aroma. J Agric Food Chem 36, 1006–1009. https://doi.org/10.1021/jf00083a025

Ferreira, V., López, R., Cacho, J.F., 2000. Quantitative determination of the odorants of young red wines from different grape varieties. J Sci Food Agric 80, 1659–1667. https://doi.org/10.1002/1097-0010(20000901)80:11<1659::AID-JSFA693>3.0.CO;2-6

Gómez-Míguez, M.J., Cacho, J.F., Ferreira, V., Vicario, I.M., Heredia, F.J., 2007. Volatile components of Zalema white wines. Food Chem 100, 1464–1473. https://doi.org/10.1016/j.foodchem.2005.11.045

Gottmann, J., Vestner, J., Fischer, U., 2023. Sensory relevance of seven aroma compounds involved in unintended but potentially fraudulent aromatization of wine due to aroma carryover. Food Chem 402, 134160. https://doi.org/10.1016/j.foodchem.2022.134160

Guth, H., 1997. Quantitation and Sensory Studies of Character Impact Odorants of Different White Wine Varieties. J Agric Food Chem 45, 3027–3032. https://doi.org/10.1021/jf970280a

Leffingwell & Associates, n.d. Odor Properties & Molecular Visualization [WWW Document]. URL http://www.leffingwell.com/esters.htm (accessed 2.1.25).

Li, H., Tao, Y.-S., Wang, H., Zhang, L., 2008. Impact odorants of Chardonnay dry white wine from Changli County (China). European Food Research and Technology 227, 287–292. https://doi.org/10.1007/s00217-007-0722-9

López de Lerma, N., Peinado, R.A., Puig-Pujol, A., Mauricio, J.C., Moreno, J., García-Martínez, T., 2018. Influence of two yeast strains in free, bioimmobilized or immobilized with alginate forms on the aromatic profile of long aged sparkling wines. Food Chem 250, 22–29. https://doi.org/10.1016/j.foodchem.2018.01.036

Martín-García, F.J., Palacios-Fernández, S., López de Lerma, N., García-Martínez, T., Mauricio, J.C., Peinado, R.A., 2023. The Effect of Yeast, Sugar and Sulfur Dioxide on the Volatile Compounds in Wine. Fermentation 9, 541. https://doi.org/10.3390/fermentation9060541

Moreno, J.A., 2005. Influencia del tipo de envejecimiento sobre el perfil aromático de vinos generosos andaluces. University of Córdoba, Córdoba.

Muñoz-Castells, R., Moreno, J., García-Martínez, T., Mauricio, J.C., Moreno-García, J., 2024. Assessing the Impact of Commercial Lachancea thermotolerans Immobilized in Biocapsules on Wine Quality: Odor Active Compounds and Organoleptic Properties. Fermentation 10, 303. https://doi.org/10.3390/fermentation10060303

Ogawa, M., Vararu, F., Moreno-Garcia, J., Mauricio, J.C., Moreno, J., Garcia-Martinez, T., 2022. Analyzing the minor volatilome of Torulaspora delbrueckii in an alcoholic fermentation. European Food Research and Technology 248, 613–624. https://doi.org/10.1007/s00217-021-03910-y

Peinado, R.A., Mauricio, J.C., Moreno, J., 2006. Aromatic series in sherry wines with gluconic acid subjected to different biological aging conditions by Saccharomyces cerevisiae var. capensis. Food Chem 94, 232–239. https://doi.org/10.1016/j.foodchem.2004.11.010

Peinado, R.A., Moreno, J., Bueno, J.E., Moreno, J.A., Mauricio, J.C., 2004. Comparative study of aromatic compounds in two young white wines subjected to pre-fermentative cryomaceration. Food Chem 84, 585–590. https://doi.org/10.1016/S0308-8146(03)00282-6

Qu, J., Chen, X., Wang, X., He, S., Tao, Y., Jin, G., 2024. Esters and higher alcohols regulation to enhance wine fruity aroma based on oxidation-reduction potential. LWT 200, 116165. https://doi.org/10.1016/j.lwt.2024.116165

Takeoka, G., Buttery, R.G., Ling, L., 1996. Odour Thresholds of Various Branched and Straight Chain Acetates. LWT - Food Science and Technology 29, 677–680. https://doi.org/10.1006/fstl.1996.0105

Zhang, S., Petersen, M., Liu, J., Toldam-Andersen, T., 2015. Influence of Pre-Fermentation Treatments on Wine Volatile and Sensory Profile of the New Disease Tolerant Cultivar Solaris. Molecules 20, 21609–21625. https://doi.org/10.3390/molecules201219791

Zhang, Y.-S., Du, G., Gao, Y.-T., Wang, L.-W., Meng, D., Li, B.-J., Brennan, C., Wang, M.-Y., Zhao, H., Wang, S.-Y., Guan, W.-Q., 2019. The Effect of Carbonic Maceration during Winemaking on the Color, Aroma and Sensory Properties of 'Muscat Hamburg' Wine. Molecules 24, 3120. https://doi.org/10.3390/molecules24173120

Zhu, L.-X., Zhang, M.-M., Shi, Y., Duan, C.-Q., 2019. Evolution of the aromatic profile of traditional Msalais wine during industrial production. Int J Food Prop 22, 911–924. https://doi.org/10.1080/10942912.2019.1612428
